# Optimal Erythrocyte Ribavirin Level to Reduce the Risk of Anemia and Obtain an Early Virological Response in Patients with Chronic Hepatitis C Caused by Genotype 1b Infection

**DOI:** 10.1155/2010/495928

**Published:** 2010-09-08

**Authors:** Rie Kubota, Takako Komiyama, Naoki Kumagai, Miyuki Kimijima, Keiko Mitsuki, Junko Uetake, Fumihiko Kaneko, Satoshi Tsunematsu, Kanji Tsuchimoto

**Affiliations:** ^1^Pharmacy Practice and Sciences, Department of Clinical Pharmacy, Center for Clinical Pharmacy and Sciences, School of Pharmacy, Kitasato University, 5-9-1 Shirokane, Minato-ku, Tokyo 108-8641, Japan; ^2^Research Center for Liver Diseases, The Kitasato Institute Hospital, Kitasato University, 5-9-1 Shirokane, Minato-ku, Tokyo 108-8642, Japan; ^3^Department of Internal Medicine, The Kitasato Institute Medical Center Hospital, Kitasato University, 6-100 Arai, Kitamoto-shi, Saitama 364-8501, Japan

## Abstract

*Aims*. To determine whether the erythrocyte phosphorylated ribavirin (RBV) level might be a useful index of EVR and risk of anemia and to determine the optimal dose of RBV in 24 patients with hepatitis C with pegylated interferon and RBV. *Methodology*. The RBV level was measured by a high-performance liquid chromatography. *Results and Conclusion*. In patients aged 50 years or over, a negative correlation (*r* = −0.548, *P* < .05) was observed between the RBV level at week 2 and rate of Hb reduction (ΔHb) at week 4. The ΔHb at week 4 was significantly greater in patients with RBV levels of ≥800 *μ*M (−25.5 ± 10.1%) than in patients with RBV levels <800 *μ*M (−15.6 ± 7.7%). None of the patients with RBV levels <600 *μ*M at week 2 achieved EVR and SVR. Thus the optimal levels of erythrocyte phosphorylated RBV at week 2 of therapy in order to achieve EVR without anemia seemed to be 600–800 *μ*M.

## 1. Introduction

The combination therapy with pegylated interferon (PEG-IFN) and ribavirin (RBV) has come to be established as the standard treatment for chronic hepatitis C. A sustained virological response (SVR) has been reported with this treatment in 30%–50% of patients with HCV genotype 1b infection, which accounts for 70% of all Japanese patients with chronic hepatitis C [1]. However, the treatment often needs to be discontinued, or the dose of RBV changed, in these patients due to the development of hemolytic anemia. On the other hand, continuous treatment is important to obtain SVR with the treatment [2].

RBV is incorporated into the cells via the equilibrative nucleoside transporter (ENT) and converted to phosphates within the cells. RBV monophosphate (RMP) and RBV triphosphate (RTP) are considered to have antiviral activity [3, 4]. In nucleated cells, the phosphorylated RBV is subsequently dephosphorylated by the dephosphorylating enzyme, and RBV is eliminated from the cells via the ENT. However, in akaryocytes such as erythrocytes, which lack the dephosphorylating enzyme, accumulation of phosphorylated RBV occurs, which diminishes the cellular ATP and alters the cellular characteristics; these changes in the characteristics of the erythrocytes activate the cell-elimination activity of the reticuloendothelial system, resulting in hemolysis [5, 6].

In this study, with the objective of reducing the adverse effects and improving the treatment completion rate in patients receiving combined PEG-IFN and RBV therapy, we attempted to evaluate whether the erythrocyte phosphorylated RBV level might be useful as an index for the rate of Early Virological Response (EVR) and SVR, the risk of anemia.

## 2. Materials and Methods

### 2.1. Subjects

Among the patients with chronic hepatitis C caused by genotype 1b infection in whom combined PEG-IFN*α*2b and RBV therapy was started, 24 patients who provided written informed consent for participation in this study were enrolled. The dosage regimen for the combined PEG-IFN and RBV therapy was determined in accordance with the standard dosing recommended for Japanese HCV patients. RBV was started at the initial dose of 600 mg/day in patients with a body weight of ≤60 kg, 800 mg/day in those with a body weight of >60 kg and ≤80 kg, and 1,000 mg/day in those with a body weight of >80 kg. In patients with no cardiovascular disease, the RBV dosage of 600 mg/day was decreased to 400 mg/day, and 800 mg or 1000 mg/day was reduced to 600 mg/day, if hemoglobin (Hb) level decreased to less than 10 g/dL; permanently discontinued the drug if Hb decreased to less than 8.5 g/dL. In those with history of stable cardiovascular disease, the dosage of RBV was decreased to 400 mg or 600 mg if Hb decreased by 2 g/dL or more during any 4-week period; permanently discontinued the drug if Hb was less than 12 g/dL after 4weeks of a reduced RBV dosage. The patients were followed up until 48 weeks after the start of the combined IFN and RBV therapy, and Hb level and HCV RNA level were examined at week 12 after the start of treatment. 

Serum HCV RNA negativity until 12 week of the therapy was defined as EVR. Additionally, serum HCV RNA negativity until 24 weeks after the therapy was completed and was defined as SVR. This study was performed with the approval of the hospital research committee, in compliance with the ethical principles laid out in the Declaration of Helsinki.

### 2.2. Measurement of the Erythrocyte Level of Phosphorylated RBV

Ten-mL samples of venous blood were obtained at 2, 4, and 8 weeks after the start of the therapy, and the erythrocyte level of phosphorylated RBV was measured by the HPLC method described by Homma et al. [7]. In this method, all phosphorylated RBV (RMP, RDP, and RTP) is converted back to RBV by treatment with an erythrocyte dephosphorylating enzyme, and the erythrocyte level of phosphorylated RBV is calculated as the difference in the RBV levels measured before and after the enzyme treatment.

### 2.3. Statistical Analysis

The changes in the Hb level were analyzed by repeated-measures ANOVA and Dunnett's test. The relationships of the RBV level to the risk of anemia and the drug efficacy were examined by Student's *t*-test, Pearson's correlation coefficient, *χ* ^2^-test, and Fisher's exact probability test. *P* < .05 was regarded as denoting clinical significance.

## 3. Results

### 3.1. Patient Characteristics and Therapeutic Course

The subjects comprised 24 patients, and their demographic characteristics are indicated in Table 1. The combined PEG-IFN and RBV therapy needed to be discontinued in 3 of the 24 patients (12.5%), and the RBV dose needed to be reduced in 7 of the patients (29.2%) due to the development of anemia (Hb ≤ 10 g/dL). One patient was discontinued the therapy due to adverse reaction except anemia. However, the conditions of RBV administration for the initial 4 weeks were not changed. None of those in whom this therapy was discontinued achieved EVR. There were no significant difference in the EVR rates between the subjects in whom the RBV dose was reduced (72.7%) and those in whom the therapy continued at the initial dose (64.0%). The dosage of PEG-IFN was 1.5 ± 0.2 *μ*g/kg in accordance with a standard regimen, and the conditions of IFN administration have not changed for 48 weeks after the combination therapy was started in the subjects except for 4 patients who discontinued the therapy.

### 3.2. Changes in the Hb Level

 The Hb levels (11.0 ± 1.3 g/dL) were significantly lower at week 4 of therapy as compared with 13.6 ± 1.3 g/dL at the start. In addition, the rate of Hb reduction [(Hb level-Hb level before administration)/Hb level before administration] at week 4 of therapy was −12.4% in those aged less than 50 years, whereas it was −21.0% in those aged 50 years or over (*P* < .05).

### 3.3. Changes in the Erythrocyte Phosphorylated RBV Level

**Figure 1:**
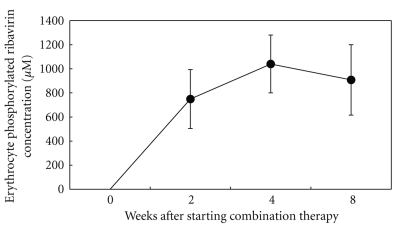
Time course of erythrocyte phosphorylated ribavirin concentration after starting PEG-IFN*α*-2b and ribavirin combination therapy.

The phosphorylated RBV and nonphosphorylated RBV levels in the erythrocytes were 749.3 ± 244.3 and 6.9 ± 3.6 *μ*M at week 2, 1039.9 ± 239.6 and 8.4 ± 7.0 *μ*M at week 4, and 907.9 ± 292.1 and 8.9 ± 8.0 *μ*M at week 8 of therapy, respectively; thus, about 99% of the RBV in the erythrocytes was phosphorylated (Figure 1).

### 3.4. Relationship between the Erythrocyte Phosphorylated RBV Level at Week 2 and the Frequency of Anemia

**Figure 2:**
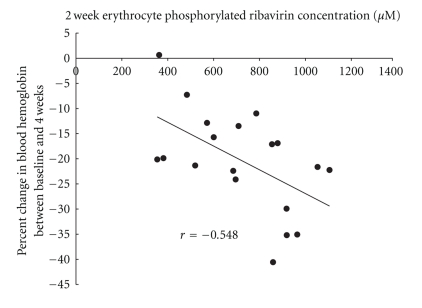
Correlation between 4-week hemoglobin reduction rate from the baseline and 2-week erythrocyte phosphorylated ribavirin concentration in patients aged 50 and over. *r* = −0.548 (*P* < .05).

**Figure 3:**
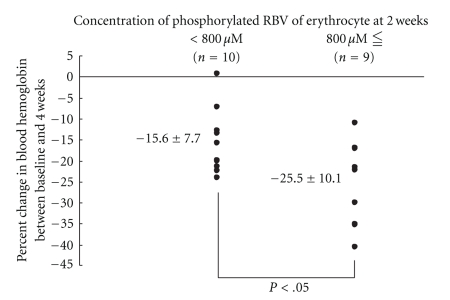
Comparison of 4-week hemoglobin reduction rate from the baseline of the patients with 2-week erythrocyte phosphorylated ribavirin concentration(<800 *μ*M) to 4-week hemoglobin reduction rate from the baseline of the patients with 2-week erythrocyte phosphorylated ribavirin concentration(800 *μ*M ≦).

The relationship between the RBV level at week 2 and the rate of reduction of the Hb level was examined in the 19 patients aged 50 years or over. There was a negative correlation (*r* = −0.548, *P* < .05) between the RBV level at week 2 and the rate of Hb reduction (ΔHb) at week 4 in the subjects in whom the dose of RBV did not reduce or the combination therapy did not discontinue until week 4 (Figure 2). As shown in Figure 3, the ΔHg at week 4 was significantly higher (*P* < .05) in those with RBV level of ≥800 *μ*M (−25.5 ± 10.1%) than in those with the level of <800 *μ*M (−15.6 ± 7.7%).

### 3.5. Relationship between the Erythrocyte Phosphorylated RBV Level at Week 2 and the EVR

The relationship between the phosphorylated RBV level at week 2 and the EVR was evaluated in 20 of the 24 patients (Four cases were excluded because of the discontinuation of the therapy). The mean RBV level at week 2 was significantly lower (*P* < .05) in the non-EVR patients (634.6 ± 236.6 *μ*M) than the EVR patients (889.7 ± 210.6 *μ*M).

As shown in Table 2, 3 cases with the phosphorylated RBV level in erythrocytes ≥800 *μ*M discontinued the combination therapy prematurely due to anemia, whereas none of 14 cases with a levels <800 *μ*M discontinued prematurely.

 There were no EVR or SVR cases (0 of 7 cases) in patients with erythrocyte phosphorylated RBV levels <600 *μ*M at week 2, whereas, in those with levels ≥600 *μ*M, 11 of 17 cases (64.7%) had EVR and 6 of 17 cases (35.3%) had SVR (*P* < .05).

Five of 7 cases (71.4%) with erythrocyte phosphorylated RBV level at week 2 of 600–800 *μ*M achieved EVR and 3 cases (42.9%) achieved SVR without development of marked anemia. None of those patients discontinued RBV due to development of anemia.

## 4. Discussion

In this study, combined PEG-IFN and RBV therapy needed to be discontinued, or the RBV dose needed to be reduced, in about 40% of the study subjects due to the development of hemolytic anemia (Hb ≤ 10 g/dL). None of the patients in whom the combined PEG-IFN and RBV therapy was discontinued by Week 12 showed SVR. However, no difference in the rate of SVR was noted between the subjects in whom the RBV dose was reduced and those in whom the treatment could be continued at the initial dose. This suggests that continuation of the combination therapy was the most important factor for achieving the desired clinical outcome. Clinically, the RBV dose reduction is performed based on the present Hb level. However, it has been noted that such dose adjustment does not effectively prevent the progression of anemia; that is, once a decrease of the Hb level has occurred, it is too late to stop the decline through RBV dose reduction, presumably because of erythropoietic delay.

In our subjects, the phosphorylated RBV level reached a steady-state by 4 weeks of RBV therapy (1040 ± 240 *μ*M), implying gradual accumulation of phosphorylated RBV. Inoue et al. [8] reported that the erythrocyte phosphorylated RBV level at the steady-state at week 4 was 1218 ± 234 *μ*M and was well correlated with Hb reduction.

However, as the Hb level had already decreased significantly by week 4, the RBV level at week 4 does not predict anemia. Therefore, we decided to evaluate whether the phosphorylated RBV level at week 2 might be useful for prediction of the subsequent development of anemia.

A close negative correlation was observed between the erythrocyte phosphorylated RBV level at week 2 and the ΔHb at week 4 in patients aged 50 years or over. In general, in elderly people, the percentage of fat cells in the bone marrow increases, and the reserve of marrow stem cells decreases with reduction of the hematopoietic mass and reduction in the ability for formation of erythroid colony-forming units [9, 10]. Additionally, RBV is known to be substantially excreted by kidney, and renal function decreases in elderly patients. Therefore, RBV accumulation is considered to be more likely to cause anemia in the elderly, especially due to the erythropoietic delay and the delay of RBV excretion. Nomura et al. reported that one of the higher risk of severe anemia was age higher than about 60 years [11].

In patients with RBV level of ≥800 *μ*M at week 2, the ΔHb at week 4 was significantly higher, and a higher percentage of patients needed discontinuation of the RBV due to the development of anemia. Thus, we recommend that the erythrocyte phosphorylated RBV level be maintained at a level of less than 800 *μ*M at week 2 for treatment safety.

The plasma RBV level has been reported not to be correlated with the SVR [12], but there have been few reports on the relationship between the erythrocyte phosphorylated RBV level and the treatment efficacy. The erythrocyte RBV level is about 150 times higher than the plasma RBV concentration, and most of the administered RBV is considered to be secreted into the urine. Of the proportion that remains in the body, most of it does accumulate as phosphorylated product within erythrocytes [13]. Furthermore, Homma et al. reported that little phosphorylated RBV existed in the plasma [13]. Since RBV taken up by cells is considered to be phosphorylated, and RMP and RTP are considered to have antiviral activities, the erythrocyte phosphorylated RBV level is a useful index for the antiviral effect of the drug. Since EVR, defined as a decrease of the HCV RNA level to 1/100 or zero at week 12 after the start of therapy, has been reported to be useful as a prognostic factor [14], we focused on not only SVR but also EVR.

In this study, the erythrocyte phosphorylated RBV level at week 2 was predictive of the EVR. Moreover, none of the patients in whom the phosphorylated RBV level at week 2 was <600 *μ*M showed both EVR and SVR. Adjustment of the RBV dose to obtain an erythrocyte phosphorylated RBV level of ≥600 *μ*M at week 2 is considered to be required to obtain an EVR. It would be necessary to have a large sample size, study of quality of life, and demonstration of better EVR and SVR in a prospective randomized trial. Recently, Fellay et al. reported that genetic variants leading to inosine triphosphatase (ITPA) deficiency protects against clinically significant decline in Hb level induced by HCV antiviral treatment [15]. We should examine the relationships between ITPA gene variants and RBV-induced anemia in Japanese populations, and evaluate the usefulness as an index to reduce the risk of anemia with erythrocyte RBV level.

## 5. Conclusion

In this study, the erythrocyte phosphorylated RBV level at week 2 is proposed as a useful indicator to determine an optimal dosage of ribavirin in patients with chronic hepatitis C under treatment with combination therapy with pegylated interferon and RBV.

## Figures and Tables

**Table 1:** Baseline characteristics of patients.

Characteristics	(*n* = 24)
Age (years)	59.9 ± 10.3
Sex (M/F)	12/12
Past treatment (Yes/No)	15/9
Body weight (kg)	57.7 ± 9.1
Ribavirin (mg/kg/day)	11.7 ± 1.5
Hemoglobin (g/dL)	13.6 ± 1.3
HCV RNA (KIU/mL)	
<100	1
100~500	3
500~850	4
850 ≦	16

Data are expressed as mean ± S.D. or number of patients

HCV: Hepatitis C virus.

**Table 2:** Comparisons of the rate in which RBV was discontinued, the rate in which RBV dosage was reduced due to development of anemia, 4-week hemoglobin reduction rate from the baseline, the EVR rate and the SVR rate of the patients with 2-week erythrocyte phosphorylated RBV concentration <600 *μ*M to 600–800 *μ*M, and 800 *μ*M ≦.

	<600 *μ*M (*n* = 7)	600–800 *μ*M (*n* = 7)	800 *μ*M ≦ (*n* = 10)
Rate of RBV discontinuation (%)	0/7 (0)	0/7 (0)	3/10 (30.0)
Rate of RBV reduction (%)	1/7 (14.3)	3/7 (42.9)	3/10 (30.0)
ΔHb by 4 weeks (%)	−13.5 ± 8.8	−15.7 ± 6.5	−23.4 ± 11.6
Rate of EVR(%)	0/7 (0)	5/7 (71.4)	6/10 (60.0)
Rate of SVR (%)	0/7 (0)	3/7 (42.6)	3/10 (30.0)

RBV: ribavirin.

ΔHb: 4-week hemoglobin reduction rate from the baseline.

EVR: Early Virological Response; Serum HCV RNA negativity until 12 weeks of therapy.

SVR: Sustained Virological Response; Serum HCV RNA negativity at 24 weeks after completed therapy.

ΔHb is expressed as mean ± S.D.
